# Dietary ω3-and ω6-Polyunsaturated fatty acids reconstitute fertility of Juvenile and adult *Fads2*-Deficient mice

**DOI:** 10.1016/j.molmet.2020.100974

**Published:** 2020-03-17

**Authors:** Wilhelm Stoffel, Inga Schmidt-Soltau, Erika Binczek, Andreas Thomas, Mario Thevis, Ina Wegner

**Affiliations:** 1 Laboratory of Molecular Neuroscience, Institute of Biochemistry, University of Cologne, 50931, Cologne, Germany; 2 CMMC (Center for Molecular Medicine), Faculty of Medicine, University of Cologne, 50931, Cologne, Germany; 3 CECAD (Cluster of Excellence: Cellular Stress Responses in Aging-Associated Diseases), University of Cologne, 50931, Cologne, Germany; 4 Institute of Biochemistry, Deutsche Sporthochschule Cologne, 50933, Cologne, Germany

**Keywords:** ω3-and ω6-PUFA deficiency, Auxotrophic *fads2−/−* mouse, Nutritional rescue of female and male fertility, Remodeling of membrane lipidomes, Reconstitution of junction systems of ovary and testis

## Abstract

**Objective:**

Polyunsaturated fatty acids (PUFAs), including essential fatty acids linoleic and α-linolenic acid and derived long chain and very long chain ω3-and ω6-polyunsaturated fatty acids, are vital structures in mammalian membrane systems and signaling molecules, pivotal in brain development, lipid, and energy metabolism and in female and male fertility during human evolution. Numerous nutritional studies suggest imbalance of PUFA metabolism as a critical factor in the pathogenesis of several human lifestyle diseases: dyslipoproteinemia, obesity, cardiovascular and neurodegenerative diseases, and infertility. The lack of unbiased animal models impedes molecular interpretation of the role of synthesized and dietary supplied PUFAs in these conditions. In this study, we used a Δ6 fatty acid desaturase (FADS2) deficient mouse mutant lacking key enzyme activity in the biosynthesis of ω3-and ω6-PUFAs from EFAs to address the molecular role of PUFAs in female and male fertility. Infertility is a hallmark of the pleiotropic but auxotrophic *fads2*−/− phenotype and is therefore helpful for stringent dietary studies on the role of individual PUFAs.

**Methods:**

**Feeding regimens:** Age- and gender-matched infertile *fads2−/−* mice were maintained on defined diets, normal diet containing essential fatty acids, and supplemented with ω6-arachidonic acid, ω3-docosahexaenoic acid, and arachidonic/docosahexaenoic acid, starting (a) after weaning and (b) initiated in 4-month-old female and male *fads2*−/− mice. Phospho- and sphingolipidomes of ovarian and testicular membrane lipid bilayers in each cohort were established and the impact on the expression and topology of membrane marker proteins, membrane morphology, germ cell development, and female and male fertility in the respective cohorts was elaborated.

**Results:**

PUFA synthesis deficiency caused a halt to folliculogenesis, atresia of oocytes, and infertility of *fads2*−/− female mice. A PUFA-deficient membrane lipid bilayer core structure led to the disassembly of the gap junction network of the follicular granulosa cells. In *fads2*−/− testis, the blood-testis barrier was disrupted and spermatogenesis arrested, leading to infertility. Sustained supply of combined AA and DHA remodeled the PUFA-deficient ovarian and testicular membrane lipidomes, facilitating the reassembly of the functional gap junction network for regular ovarian cycles and the reconstitution of the blood-testis barrier in Sertoli cells, reconstituting fertility not only in developing newborns, but surprisingly also in adult infertile *fads2*−/− mice.

**Conclusions:**

These findings demonstrate the previously unrecognized membrane structure-based molecular link between nutrient ω3-and ω6-PUFAs, gonadal membrane structures, and female and male fertility and might foster studies of the pivotal role of dietary PUFAs in human fertility.

## Highlights

• PUFA-depletion disrupts membrane lipid scaffolds of ovarian GJ- and TJ-complexes of the testicular BTB
• Nutrient AA/DHA reconstitute the gonadal membrane bilayer architecture in auxotrophioc *fads2-/-* mice
• AA/DHA replenished lipid-bilayers promote the assembly of follicular GJ- and BTB-protein complexes in *fads2-/-* mice
• Nutrient AA/DHA release arrest of oo- and spermatogenesis, restoring fertility of newborn and adult *fads2-/-* mice

## Graphical abstract

**Figure:**
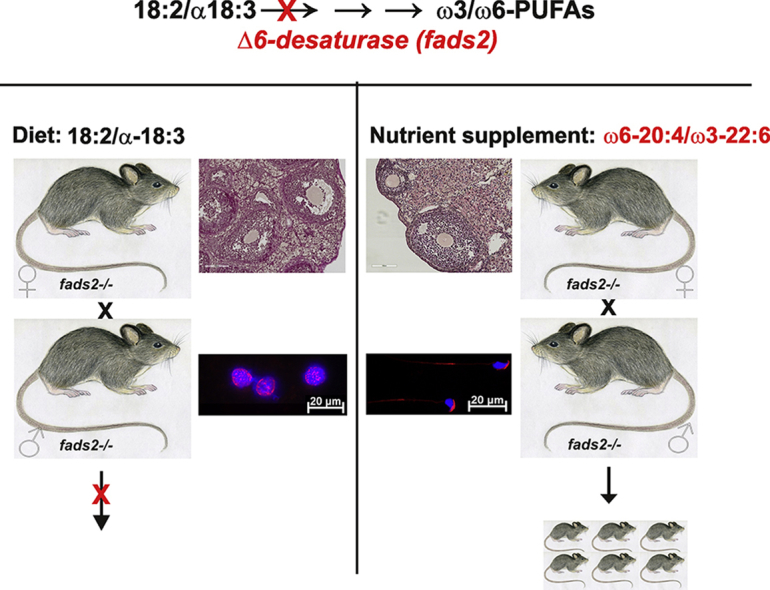

## 1 Introduction

Polyunsaturated fatty acids (PUFAs) include the essential fatty acids (EFAs), ω6-linoleic acid (18:2^9,12^), and ω3-α-linolenic acid (18:3^9,12,15^), which are indispensable for cell viability. They are utilized as precursors for the synthesis of long chain (LC-PUFAs) (<C24) and very long chain PUFAs (VLC-PUFAs) (C24–C36) with two to six cis double bonds [1]. The initial bottleneck reaction of PUFA biosynthesis is the desaturation of EFAs by Δ6 fatty acid desaturase (FADS2). Further transformation by alternating chain elongation by elongases (ELOVLs) and Δ5 desaturation by FADS1 leads to the ω3-and ω6-LC-PUFA families, main representatives of which are ω6-arachidonic (20:4^5,8,11,14^, AA) and ω3-docosahexaenoic acid (22:6^4,7,10,13,16,19^, DHA) [2]. Homeostasis of the cellular pool of EFAs and derived LC-PUFAs is maintained solely by nutritional sources.

PUFAs are structural constituents of the hydrophobic core of membrane phospholipid bilayers, which due to their inherent biophysical properties provide the optimal scaffold for integral membrane proteins such as receptors, transporters, channel proteins, adhesion proteins, and enzymes. In addition, PUFAs serve as precursors of a variety of lipophilic ligands in divergent signaling pathways.

Imbalance of the ω3/ω6-PUFA ratio in the current Western diet is regarded as a critical epigenetic factor in the pathogenesis of several lifestyle diseases: obesity and cardiovascular diseases, neurodegeneration, and brain development. A plethora of proposed pathologies has been addressed in numerous studies in model systems, notably rodents [3], chickens [4], rhesus monkeys [5], and human infants [[6], [7], [8]]. However, the lack of unbiased model systems has left the understanding of the molecular level of the systemic and cell-specific complex functions of PUFAs largely enigmatic. The PUFA synthesis-deficient auxotrophic *fads2*−/− mouse mutant is beneficial for controlled nutritional studies elaborating the systemic physiological and cell-specific roles and molecular impact of EFAs, ω3-and ω6-LC-, and VLC-PUFAs [9,10] in the proposed pathologies.

Infertility is a hallmark of FADS2 deficiency [9]. The ovarian cycle is regulated by endocrine and intrinsic signaling in the gap junction (GJ) and tight junction (TJ) network of granulosa cells (GCs) and between oocytes and GCs. The entire differentiation process of male germ cells during spermatogenesis from diploid type A spermatogonia to haploid spermatids proceeds between supporting highly polarized Sertoli cells (SCs) under the hormonal control of pituitary and Leydig cells. TJ, ectoplasmic specialization, and GJ-protein complexes integrated into the plasma membrane form the impermeable blood-testis barrier (BTB) between adjacent SCs (Mruk and Cheng, 2004, 2010; Carette et al., 2010; Pelletier, 2011; Franca et al., 2012; Kaur et al., 2014; Stanton, 2016), which during the seminiferous epithelial cycle is transiently disassembled for apical progression of preleptotene and leptotene spermatocytes from the basolateral compartment across the barrier and reassembled [11,12].

In this study, we utilized the unbiased *fads2−/−* mutant as a platform to explore the underlying molecular basis of infertility in *fads2−/−* mice. The hydrophobic diacylglycerol (DAG) core of phospholipid bilayers of membranes of GCs in ovarian follicles and phospho- and sphingolipidome of SCs and GCs of the testicular tubular system are deprived of LC- and VLC-PUFAs. We first modified the lipidomes of the ovary and testis in cohorts of newborn *fads2−/−* mice starting at p21 after weaning the sustained supply of diets supplemented with a) EFAs (nd-*fads2−/−*), b) ω6-AA (AA-*fads2−/−*), c) ω3-DHA (DHA-*fads2-/*), and d) equimolar ratio of ω6-AA and ω3-DHA (AA/DHA-*fads2−/−*) to follow the gonadal development, folliculogenesis, and spermatogenesis after maturation.

We then remodeled the lipid bilayers of the ovaries and testes of adult 4-month-old infertile *fads2−/−* mice using the nutritional supply of EFAs, AA, DHA, or AA/DHA to assess the structural essentials for the initiation of spermato- and folliculogenesis and AA/DHA replenishment of membrane lipid bilayer structures not only of *fads2−/−* testes but also *fads2−/−* ovaries fully reconstituted fertility and fecundity.

We elaborated a first molecular view of the role of dietary AA and DHA as indispensable constituents of the membrane lipid bilayer for the assembly and dynamics of the cellular and intercellular junction systems of granulosa cells of ovary follicles during folliculogenesis and of SCs during spermatogenesis.

## 2 Materials and methods

### 2.1 Mouse line

The *fads2−/−* mouse line was developed in our laboratory [9] and back-crossed into and maintained on a C57BL/6 background. The animals were housed under specific pathogen-free conditions. Control and *fads2−/−* mice were obtained from heterozygous *fads2+/−* breeding. The light/dark cycle was 12 h/12 h. The animal studies reported in this manuscript followed the ARRIVE Guidelines [13]. Animal breeding and test protocols followed the principles and practices outlined in the Guide for the Care and Use of Laboratory Animals. They were approved by the Institutional Animal Care and Use Committee of the University of Cologne and with permission of the State Agency for Nature, Environment and Consumer Protection, North Rhine-Westphalia.

The mice were genotyped by PCR analysis of the tail DNA. Cohorts of gender- and weight-matched control and *fads2−/−* male and female mice were used in this study. The normal, basic Altromin diet #1310 (Altromin, Lage, Germany) (nd) contains two EFA 18:2 and α-18:3 to prevent EFA deficiency. The nd diet was supplemented with ω6-20:4 (AA), ω3-22:6 (DHA), and ω6-20:4/ω3-22:6 (AA/DHA) for the transformation of the nd-*fads2−/−* cohorts into AA-, DHA-, and AA/DHA-*fads2−/−* mouse lines. Arachidonic acid (AA) was administered as ARASCO and docosahexaenoic acid (DHA) as DHASCO triglyceride, with 50% 20:4 (AA) and 22:6 (DHA), respectively, as single PUFA in the normal diet (nd). Table SI1 summarizes the GC/MS analysis of the fatty acid composition of the diets used in the sustained long-term feeding experiments.

We applied standardized feeding regimens of these diets a) to cohorts of newborn *fads2−/−* mice starting after weaning, p21, and sustained during lifespan. b) The feeding regimen was started in the cohorts of infertile adult *fads2−/−* mice that had been on nd diet until the onset of the sustained AA/DHA dietary regimen at the age of 4 months. Fertility recovered within 8 weeks.

### 2.2 Lipidome analysis

Total lipids of the pooled ovaries and testes cohorts (n = 5) of the control and *fads2−/−* female and male mice were extracted and separated by HPTLC for MS/MS and GC/MS analysis as described under SI.

### 2.3 Gene expression analysis via qRT-PCR

RNA was isolated from the control and *fads2−/−* ovaries and testes of nd-, AA-, and AA/DHA-wt and *fads2*−/− mice and using TRIzol (Invitrogen). Then 10 μg of total RNA was reverse-transcribed using a Transcriptase kit (Life Technologies, Darmstadt, Germany). Primer pairs used in the quantitative PCR reactions are listed in Supplemental Table 2. Hgprt was used as an internal standard. The qRT-PCR reactions were conducted with an ABI Prism 7900HT employing a 96-well format and Fast SYBR Green Master Mix (Applied Biosystems) following the manufacturer's protocol. Data analysis was performed using the 2-ΔΔCt method.

### 2.4 Protein analysis

Protein analysis of lysates of the control and *fads2*−/− ovaries and testes via Western blotting is described under the SI.

### 2.5 Sperm analysis

Epididymes of adult male nd, AA, DHA, and AA/DHA mice were isolated for sperm count, motility and purity check, and immunohistochemistry as described in the SI.

### 2.6 Ultrastructure analysis

The ovaries and testes from the control and *fads2−/−* mice were perfused with PBS and fixed for 1 h at 4 °C with 2% glutaraldehyde, 2% PFA, and 0.2% picric acid in 0.1 M cacodylate buffer at a pH of 7.35. The fixation buffer was removed and the tissue specimen washed 3× with 0.1 M cacodylate buffer at a pH of 7.35, post-fixed in 1% OsO4 solution for 1 h, and stained in 1% uranyl acetate for 1 h at room temperature. After dehydration, the specimens were embedded in Araldite (Serva, Heidelberg, Germany). Ultra-thin sections (70 nm) were stained with uranyl acetate and lead citrate and examined via EM (Zeiss 902A, Zeiss, Oberkochem, Germany). Semi-thin sections (1 μm) were stained with methylene blue for light microscopy.

### 2.7 Statistical analysis

The results are expressed as mean ± SEM. The statistical significance of the differences between the individual experimental groups was calculated via the unpaired *t*-test using GraphPad Quick Calcs t-test calculator. P values of ≤0.05∗, ≤0.01∗∗, and ≤0.001∗∗∗ were considered significant.

## 3 Results

### 3.1 Reconstitution of the disrupted granulosa cell network in the follicles of the *fads2−/−* ovaries remodeled by nutrient AA/DHA

**Figure 1:**
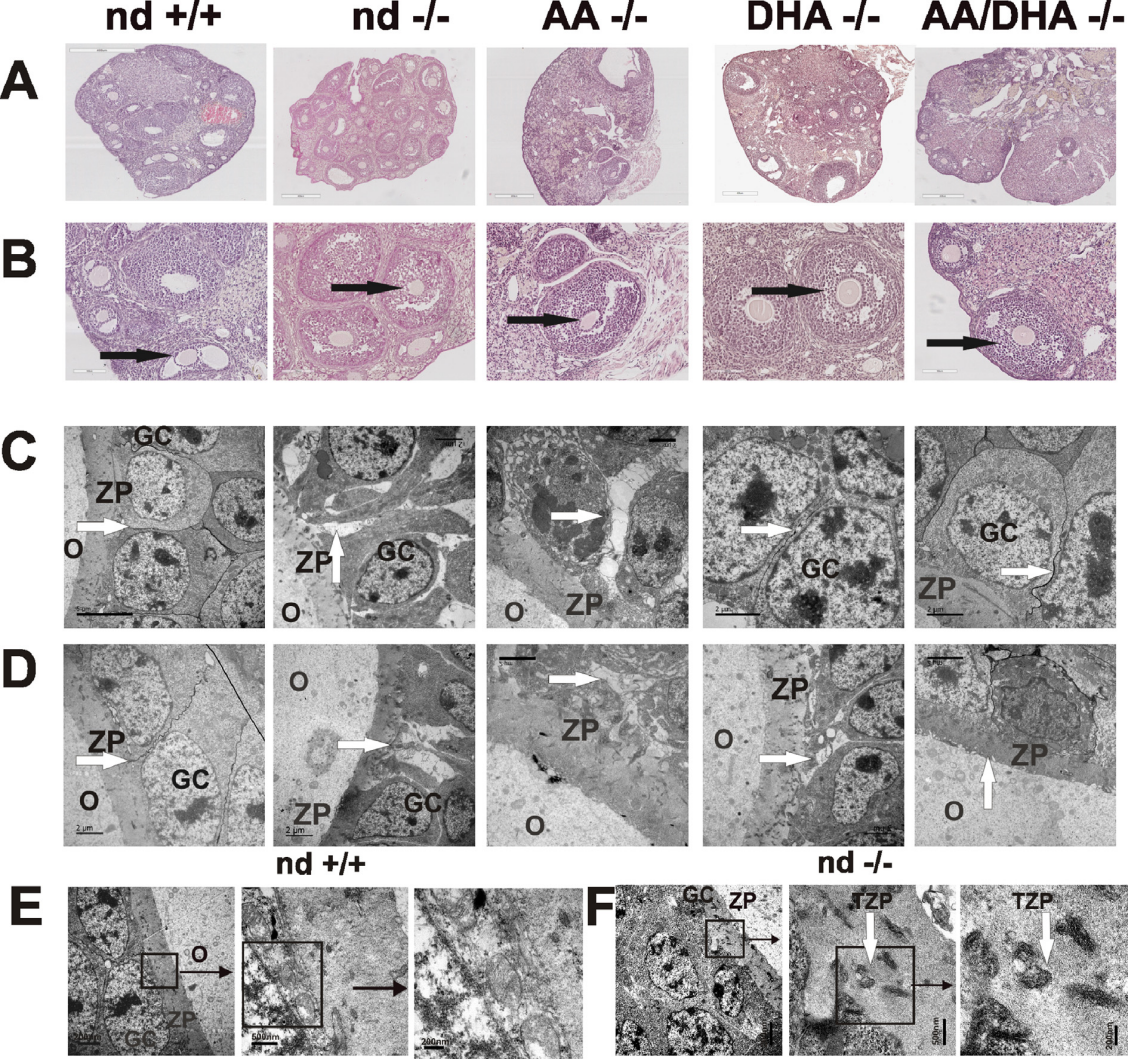
Reconstitution of the ovarian cycle in adult 6-month-old infertile *fads2*−/− females via an AA/DHA diet after a feeding period of 2 months. (A and B) HE-stained sections and (C and D) EM images of the GCs, zona pellucida (ZP), and ovum (O) of the control, nd-, AA-, DHA- and AA/DHA-*fads2−/−* ovaries at two magnifications. Arrows: Disrupted and reconstituted GC adhesion, ZP and transzonal projections (TZPs), and EM images of ZP in the (E) control and (F) nd-*fads2−/−* ovaries at two magnifications.

The morphology of the ovaries of the infertile nd-*fads2−/−* adult females and ovaries of *fads2*−/−cohorts under AA, DHA, and AA/DHA sustained diets is shown in Figure 1 A-F. HE-stained sections revealed the recovery of the *follicular phase*, *ovulation*, and *luteal phase (ovarian cycle)* by nutrient AA/DHA.

Next we applied transmission electron microscopy, which displayed disrupted granulosa cell layers and widened intercellular spaces between dissociated GC columns and malformation of the zona pellucida (ZP) follicles of nd-*fads2−/−* and AA-*fads2−/−* ovaries. The nd-*fads2−/−* female ovaries responded to sustained DHA and combined AA/DHA diets with tightly packed GC layers and ZP structures as shown in Figure 1C–F.

**Figure 2:**
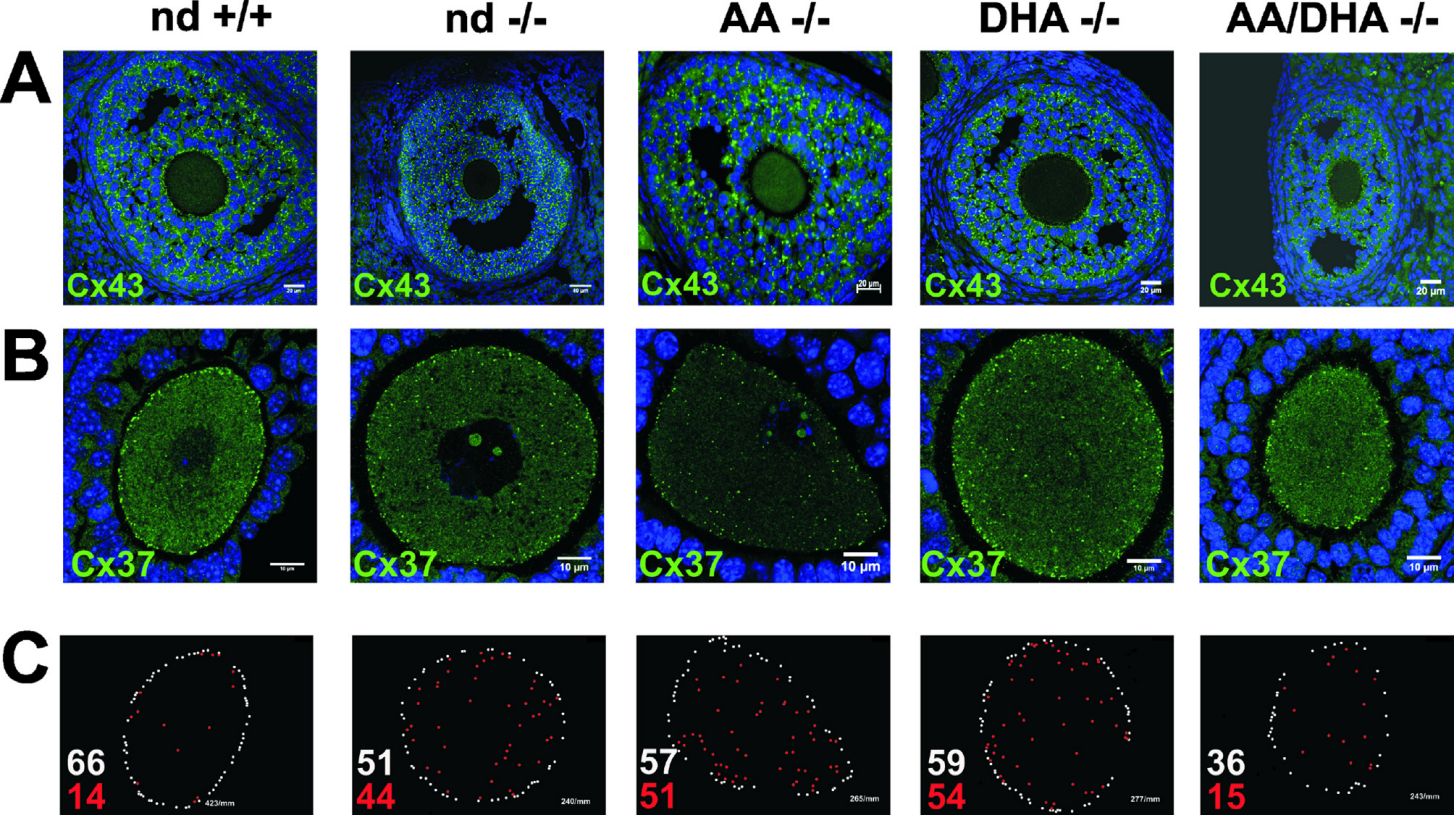
Reconstitution of the GJ network of the GCs and GC ovum connectivity in the ovaries of the AA/DHA-*fads2−/−* mice aged 6 months. IHC of sections of the control, nd-, AA-, DHA-, and AA/DHA-*fads2−/−* ovaries stained with (A) anti-connexin (Cx) 43 and (B) Cx37 antibodies. (C) Semi-quantitative evaluation of Cx37 antigen lined up at the interphase between the ovum and granulosa cells (white dots) and dislocated (red dots) in the nd- and AA- and partially in the DHA-*fads2−/−* ovaries.

Immunohistochemical studies displayed the topology of marker proteins of GJ and TJ in the control, nd-, AA-, DHA-, and AA/DHA-*fads2−/−* ovaries as shown in Figure 2A–C. Anti-Cx43 antibody was used to trace channel-forming pannexins between the plasma membranes of the adjacent GCs and connexons. Cx43 topology revealed the reconstitution of the GJ network between tightly packed GC layers in the DHA- and AA/DHA-*fads2−/−* follicles, but a disrupted GC network in the nd- and AA-*fads2−/−* follicles as shown in Figure 2A. Cx37 topology was confined to the GC zona pellucida (ZP) ovum interphase in follicles of the control and AA/DHA-*fads2−/−* ovaries, but dissipated from the basal compartment of the polarized GCs adherent to the ZP of the nd- and AA-*fads2−/−* follicles as shown in Figure 2B. We then semi-quantitatively evaluated the ratio of Cx37 localized at the oocyte periphery and scattered over the ovum, which further indicated disrupted intercellular connexons at the GC-ovum interface as shown in Figure 2C. These different experimental approaches clearly support the observation that the AA/DHA diet remodeled the granulosa cell membrane lipidome to assemble a regular GJ network.

### 3.2 Rescue of spermatogenesis in the infertile *fads2*−/− mice via the AA/DHA diet

**Figure 3:**
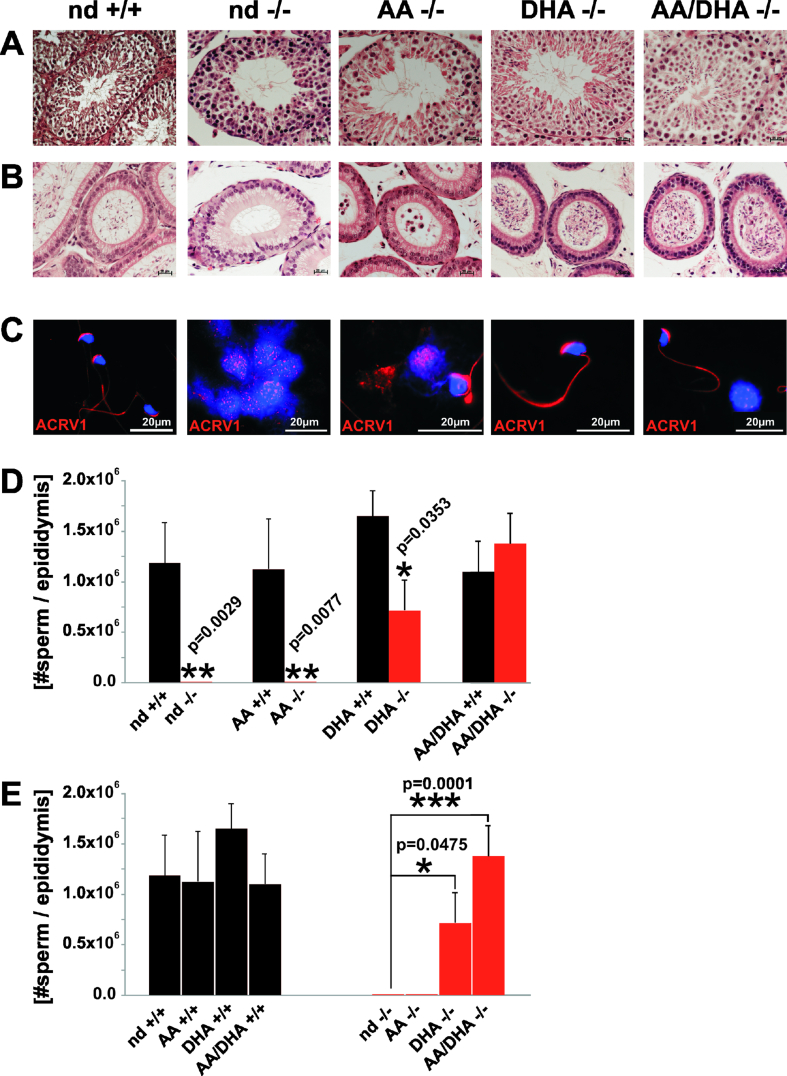
The AA/DHA diet abolished azospermia of the 6-month-old adult nd-*fads2−/−* males. HE stained sections of the control, nd-*,* AA-, DHA-, and AA/DHA-*fads2−/−* mice (6 months) of the (A) testis, (B) epididymis, and (C) IHC image of epididymal smear (washout) stained with anti-acrosomal vesicle protein 1 (ACRV1) antibody. (D and E) Sperm counts in epididymal washouts of the nd-, AA-, DHA- and AA/DHA-, and control *fads2−/−* males (6 months).

The mature nd-*fads2−/−* males demonstrated azospermia. Sustained feeding of EFA (nd) and AA diets to the auxotrophic *fads2−/−* males was inefficient to promote transitions of round cell spermatocytes I into first meiosis and spermiogenesis (Figure 3A,B). Mature spermatozoa were missing in the tubular system and epididymis (Figure 3A,B). The DHA diet induced spermato- and spermiogenesis in the DHA-*fads2−/−* males. However, only the AA/DHA (1:1 M ratio) diet fully restored male spermatogenesis in the ω6/ω3-*fads2−/−* cohorts.

IHC of nd- and AA-*fads2−/−* epididymal washout smears visualized spreading of testis-specific differentiation antigen acrosomal vesicle protein 1 (ACVR1) reactive antigen over the entire cytoplasm of the spermatocytes. ACVR1 was concentrated to the cap structure associated with the acrosomal membrane in the head of the spermatozoa of the DHA- and AA/DHA-*fads2−/−* epididymis as shown in Figure 3C. Sperm counts in the epididymal washouts of the AA/DHA-*fads2−/−* males exceeded that of the DHA-*fads2−/−* males as shown in Figure 3D,E.

The DHA-*fads2*−/− males showed reduced spermatogenesis, and in fertilizing the controls, the females had only a low number of progeny, three pregnancies out of 12 matings with 2, 3, and 8 offspring. The DHA*-fads2−/−* females were infertile when mated with the control males (n = 12).

**Figure 4:**
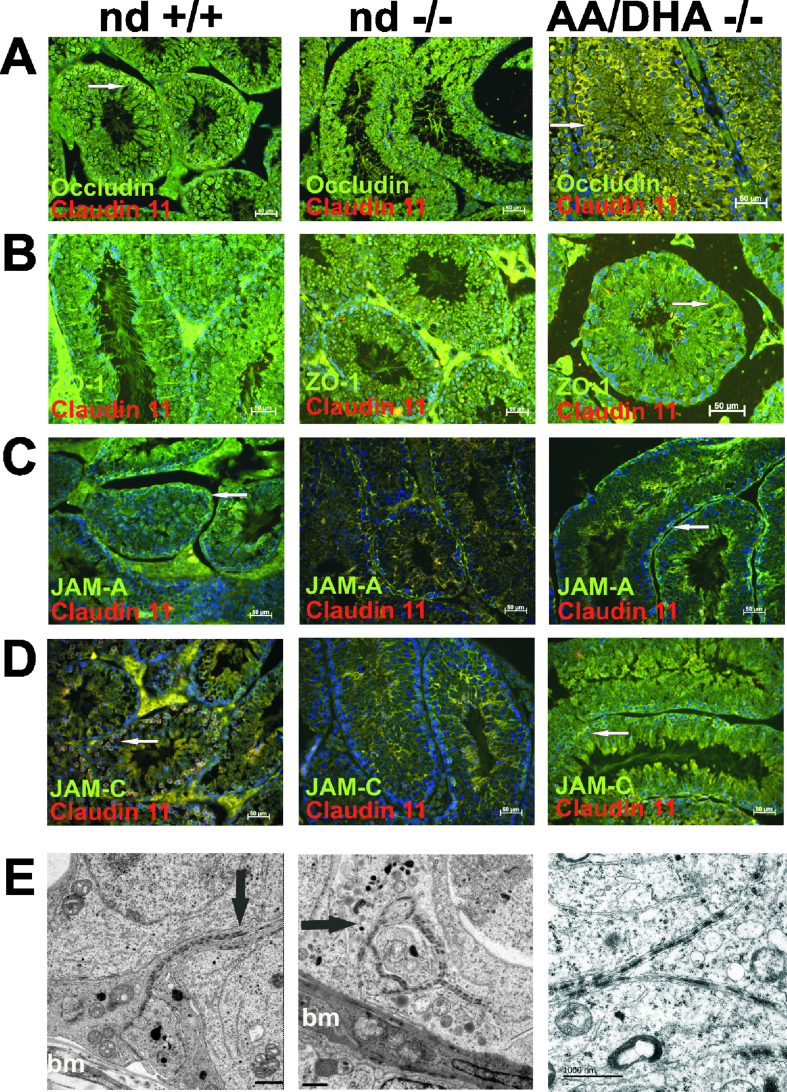
Reconstitution of polarity of Sertoli cells in adult 6 month old infertile *fads2*−/− males after a feeding period of 2 months. IHC of sections of control, nd-, and AA/DHA-*fads2−/−* testis. Images of double-stained sections using anti-Claudin 11 (Cy3) merged (A) anti-Occludin, (B) anti-*ZO1*, (C) anti-*JamA*, and (D) anti-*amC*J antibodies labeled with FITC-conjugated 2 antibody. (E) Reconstitution of the BTB in the AA/DHA testes. EM of lanthanum perfused nd control, nd, and AA/DHA-*fads2−/−* testes. Arrows: Lanthanum–impermeable junction domains in the control and AA/DHA-*fads2−/−* and lanthanum diffusion in the nd-*fads2−/−* testes (bm, basal membrane).

We determined the plasma concentrations of the gonadal steroids testosterone and progesterone in the cohorts of the nd-, AA-, DHA- and AA/DHA-*fads2−/−* mature mice (n = 10 each) using LC-MS as shown in Figure SI2. No significant differences in the plasma levels of the gonadal steroids were measured. This excluded a missing endocrine support causing interrupted gametogenesis. The well-developed Leydig cell clusters, as shown in Figure 4B, provide normal stimulation of adult Sertoli cells and seminiferous tubuli.

### 3.3 Reconstitution of the disrupted blood-testis barrier of *fads2−/−* Sertoli cells via the AA/DHA diet

We immunohistochemically visualized the topology of TJ-specific marker proteins Occludin, Claudin 11, *ZO1*, *amA*J, and *JamC* forming the BTB of the seminiferous tubular system in the control males. The images in Figure 4A–D displays these antigens displaced over the basolateral and apical domains of the SCs in the nd-*fads2−/−* testis. These markers reassembled in the basolateral plasma membrane domain of SCs in *fads2−/−* testis following the sustained AA/DHA dietary regimen.

Transmission electron microscopy (EM) of lanthanum perfused control and AA/DHA-*fads2−/−* testes revealed a regular ultrastructure of the junction domains between the basolateral compartments of the adjacent SCs forming the BTB. Diffusion of lanthanum beyond the basolateral area of the BTB in nd- and AA-*fads2−/−* testis documented the perturbed TJ, GJ, and desmosome structures (Figure 4E).

### 3.4 Nutrition-dependent modification of the PUFA patterns of the phospho- and sphingolipidomes in the ovaries and testes of the infertile *fads2−/−* mice

We next investigated the structural modification of the PUFA patterns of the phospholipidomes in the ovaries and testes of the control and *fads2−/−* mice under the respective sustained dietary regimen.

Phospholipid and sphingolipid classes of the total lipid extracts of the ovaries and testes of the adult nd-, AA-, DHA-, and AA/DHA-*fads2−/−* mice were separated by HPTLC and characterized by MS/MS analysis and their fatty acid substituents identified and quantitated as methyl esters (FAMEs) via GC/MS.

**Figure 5:**
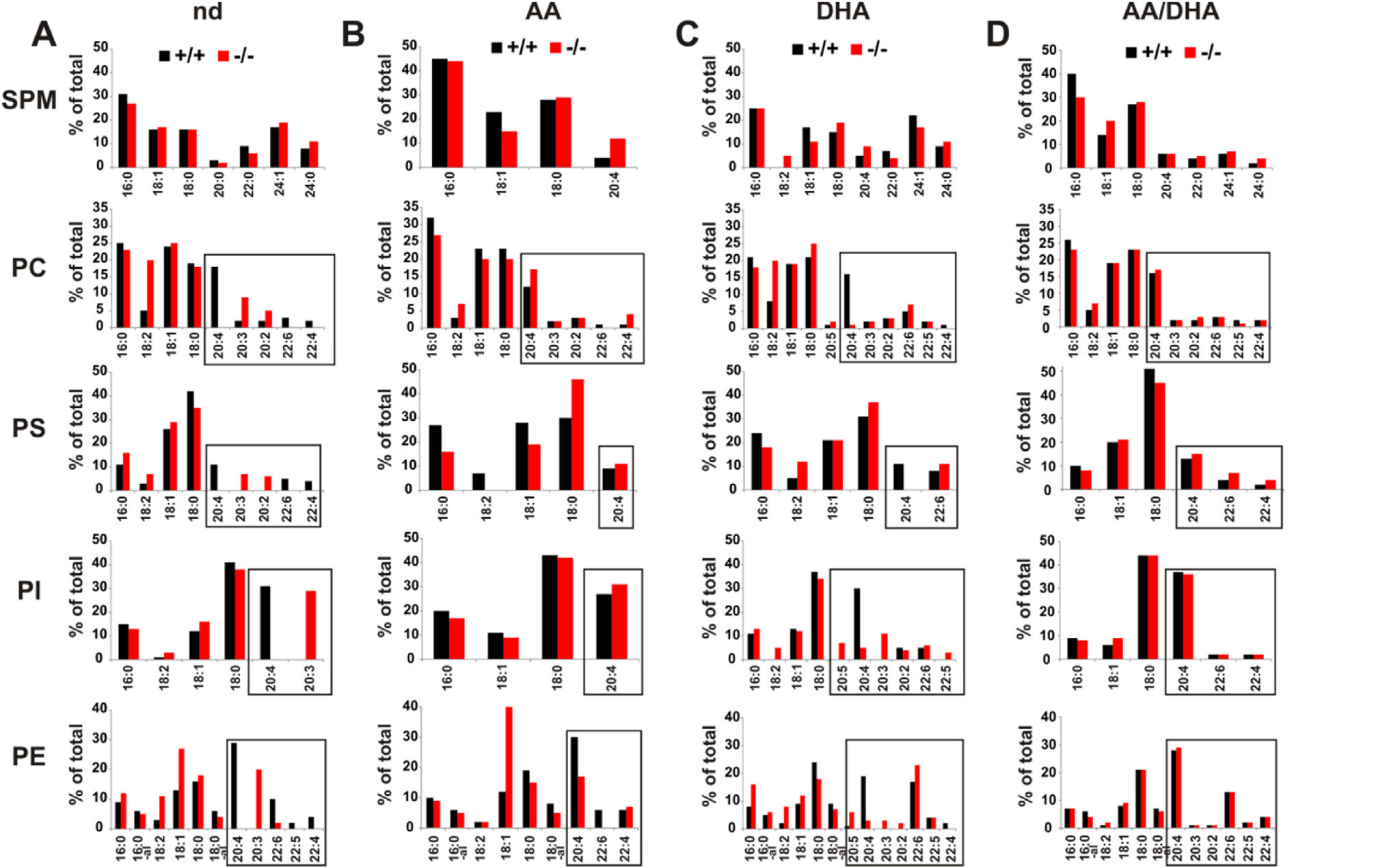
Reconstitution of the ovarian fatty acid profiles of the 6-month-old *fads2−/−* mice on the sustained ω6/ω6-PUFA diet after a feeding period of 2 months started after weaning at p21. Fatty acid patterns of the phospholipid classes of the ovaries of the control and *fads2−/−* mice on (A) nd, (B) AA, (C) DHA, and (D) AA/DHA diets. The dynamics of PUFA replenishment of the PL classes are highlighted in the boxes.

Figure 5 displays the fatty acid profiles of the lipid classes of the ovaries of the cohorts of the *fads2−/−* mice under the dietary regimens. The PUFA patterns are shown in the bar diagrams.

The PUFAs in the DAG core of all of the PL classes were stoichiometrically replaced by surrogate eicosatrienoic acid ω6- 20:3^5,11,14^. The AA, DHA, and AA/DHA diets rapidly substituted 20:3^5,11,14^ in the modified phospholipid classes.

**Figure 6:**
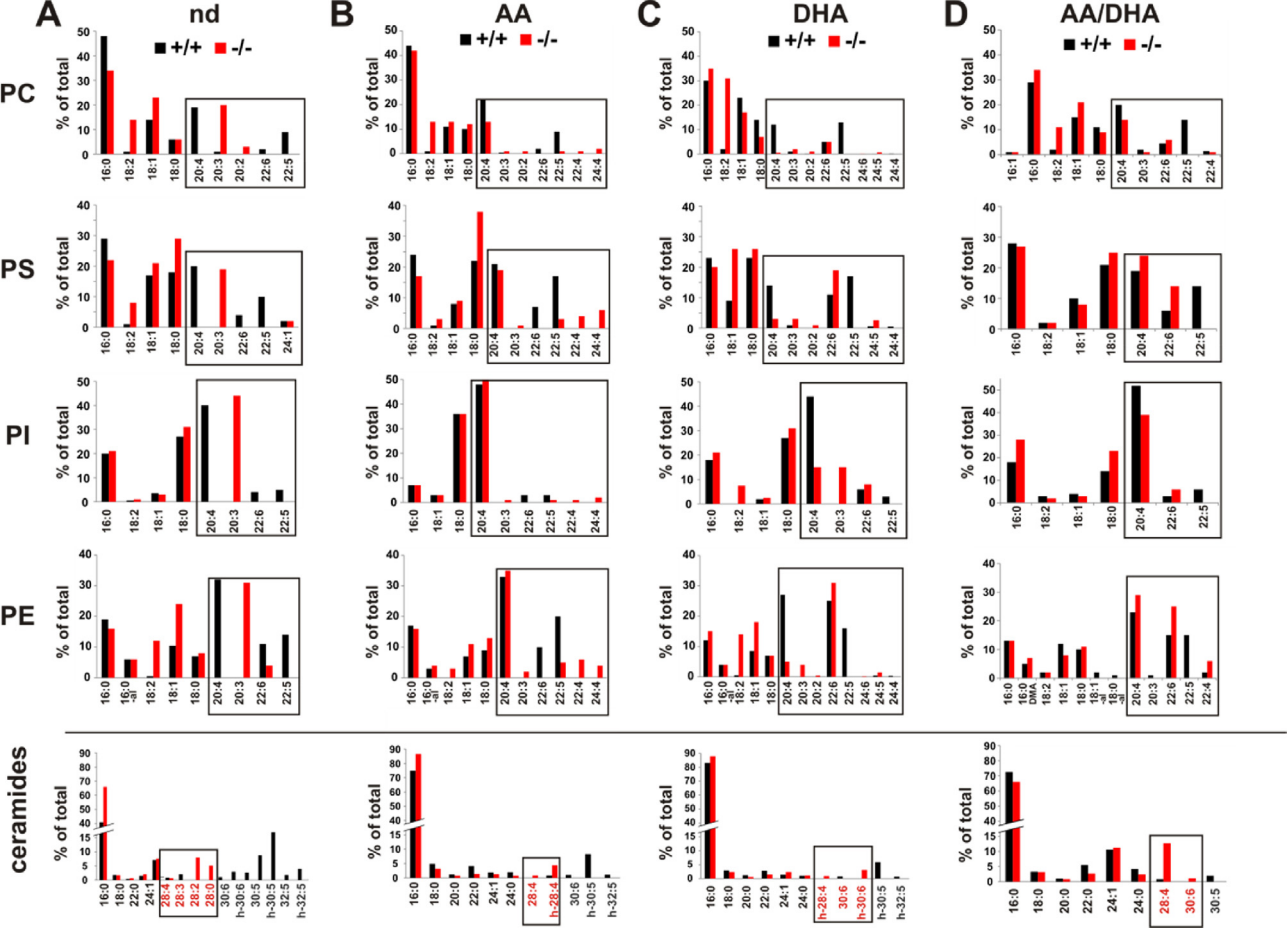
Remodeling of the fatty acid profiles of the phospholipidomes of the testes of the *fads2−/−* mice on the sustained ω6/ω6-PUFA diet after a feeding period of 2 months started after weaning at p21. Fatty acid patterns of the phospholipid classes of the testes of the control and *fads2−/−* mice on (A) nd, (B) AA, (C) DHA, and (D) AA/DHA diets. The dynamics of PUFA replenishment of the PL classes, the surrogate function of ω6-20:3^5,11,14^ in nd-*fads2−/−* testes, and the modifications in VLC-PUFA substituted ceramide species in the nd-*fads2−/−* testes on the respective diets are highlighted in the boxes.

ω3-22:5, present in the PC, PS, PI, and PE of the control testes as the most abundant VLC-PUFA overriding 22:6 concentration, was generated neither upon AA, DHA, nor AA/DHA dietary supply. The ω6-AA supply led to chain-elongated ω6-22:4 and ω6-24:4, which were absent in the control mice. Ceramide and sphingomyelin species in the sphingolipidomes of the testes of the control mice contained ω6-30:5 and ω6-h-30:5 as dominant substituents, whereas the nd-*fads2−/−* and AA-*fads2−/−* testes were devoid of VLC-PUFAs (>C28). DHA supplementation led to the synthesis of h-30:6-substituted ceramide species as the dominant VLC-PUFAs in the DHA-*fads2−/−* testes. The lipid bilayer of the *fads2−/−* testes reconstituted by the AA/DHA diet contained predominantly ω6-28:4 as shown in Figure 6 and Fig. SI1.

### 3.5 Impact of the PUFAs on the regulation of the gene and protein expression in folliculogenesis and spermatogenesis of the *fads2−/−* mice

**Figure 7:**
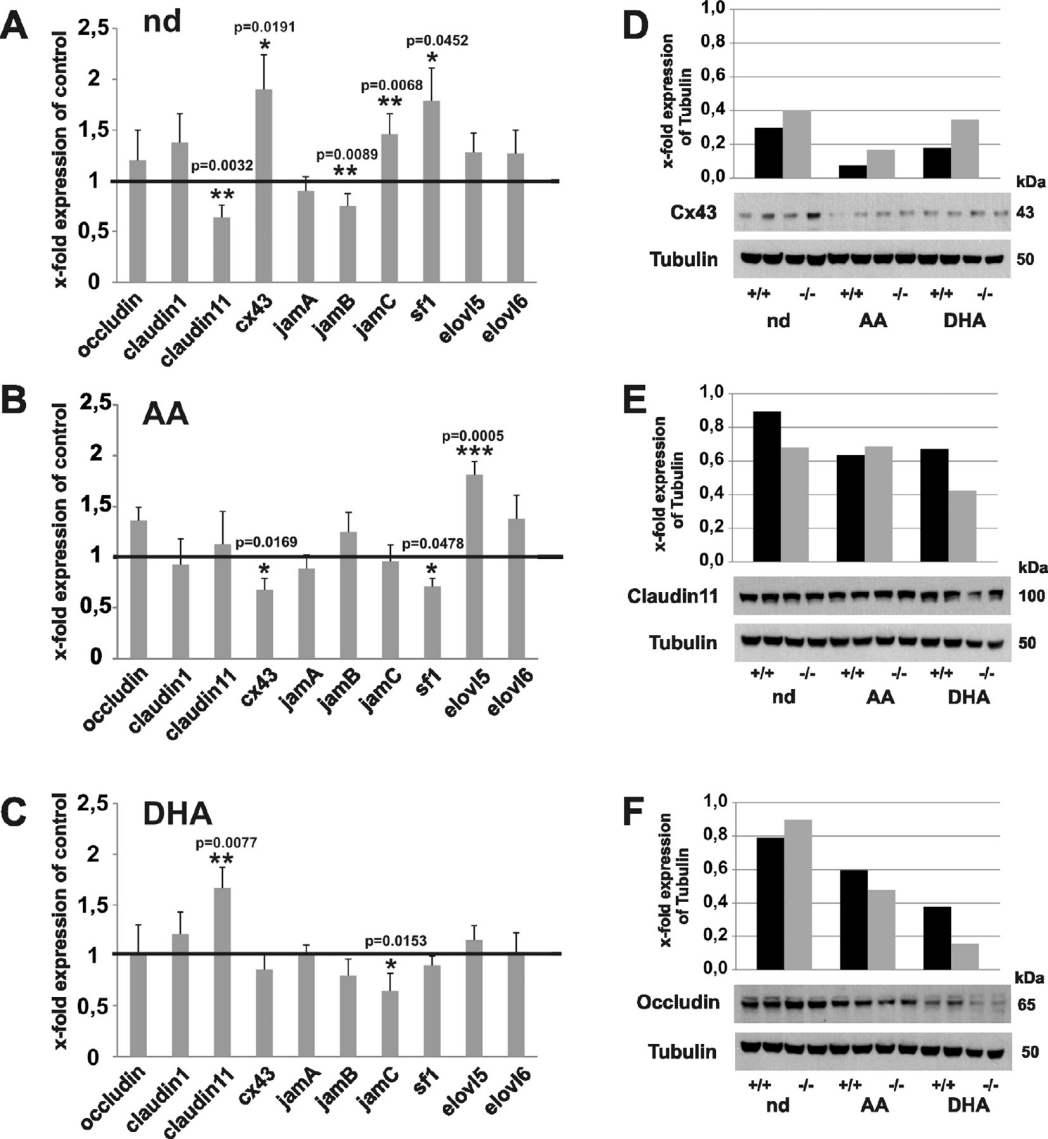
Gene expression of the GJ and TJ proteins of the adult, 6-month-old wt, nd, AA, and DHA ovaries determined via real-time PCR and Western blotting hybridization. (A–C) Fold-expression of occludin, claudin 1, claudin 11, *cx43*, *jamA*, *jamB*, *jamC*, *sf1*, *elovl5*, and *elovl6* of the (A) nd-, (B) AA-, and (C) DHA-*fads2−/−* ovaries normalized to wt-nd, AA, and DHA. (D–F) Western blotting of junction complex proteins in lysates of the ovaries of the nd-, AA-, and DHA-*fads2−/−* mice using (D) anti-Cx43, (E) anti-Claudin 11, and (F) anti-Occludin antibodies.

Using gene expression via real-time PCR, the transcript levels in the ovaries of the nd-, AA-, and AA/DHA-*fads2-/-* females were compared to the cohorts (n = 5) of age- and gender-matched weight littermates of *fads2+/−* breeding. Significantly elevated levels of transcripts of *cx43* and steroidogenic factor (*sf1*) were measured via real-time PCR in the nd-*fads2−/−* ovaries, but there was downregulation in the *cx43* expression in the AA-*fads2−/−* ovaries (Figure 7A,B). The expression of claudin 11 was elevated in the DHA-*fads2 −/−* mice ovaries (Figure 7C). Western blotting analysis of the protein lysates of the ovaries displayed increased GJ-specific Cx43 expression in the nd-, AA-, and DHA-*fads2−/−* ovaries, (Figure 7D), but reduced synthesis of Claudin 11 in the nd- and DHA*-fads2−/−* ovaries and Occludin in the DHA*-fads2−/−* ovaries (Figure 7F).

**Figure 8:**
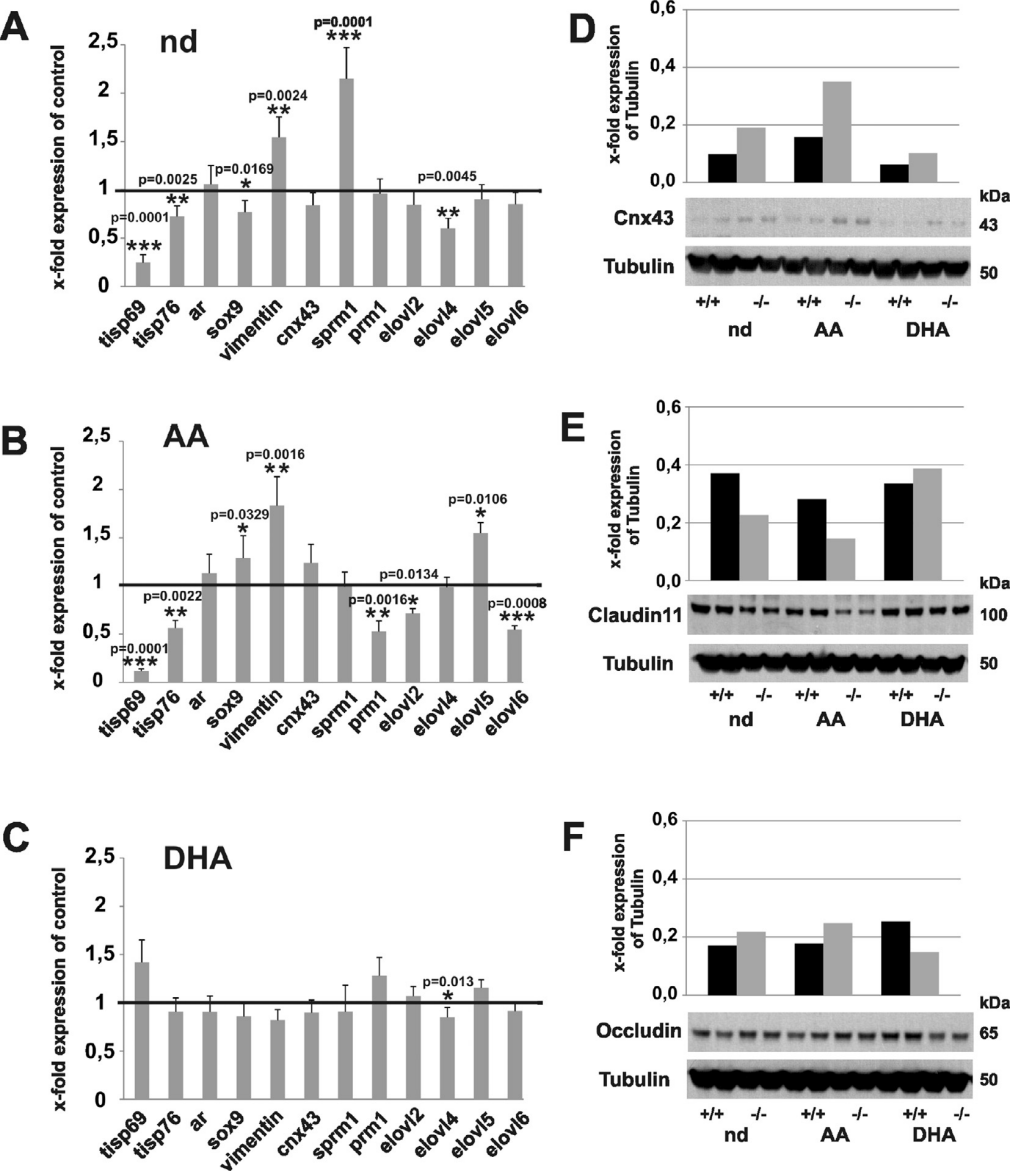
The expression of spermatogenesis-specific genes in the adult 6-month-old mice. (A–C) Real time PCR of *tisp69*, *tisp76*, ar, *sox9*, vimentin, *cx43*, *sprm1*, *prm1*, and *elovl2*, *4*, *5*, and *6* in the cRNA of the testis. Fold expression of control in the (A) nd-, (B) AA-, and (C) DHA*-fads2−/−* males normalized to wt-nd, -AA, and -DHA. (D–F) Western Blotting analysis of the junction complex proteins in lysates of the nd-, AA-, and DHA*-fads2−/−* testes using (D) anti-Cx43, (E) anti-Claudin 11, and (F) anti-Occludin antibody.

The qRT-PCR of cRNA of the nd-*fads2−/−* testes revealed elevated stationary RNA concentrations of transcription factor *sprm1*, a marker in differentiating haploid spermatids as shown in Figure 8. The expression of *tisp69* and *76*, two sperm tail-specific fibrous structural elements, was downregulated in the nd- and AA-*fads2−/−* mice and *prm1* (protamine 1) in the AA-*fads2−/−* mice (Figure 8A,B). *Prm1* condenses sperm DNA into a highly compacted complex during the haploid phase of spermatogenesis. Intermediate filament vimentin expression, a major component with vital function in contractility and cell migration, was elevated in the nd- and AA-*fads2−/−* testes but remained stable in the DHA-*fads2−/−* mice testes throughout the lifetime. The *elovl2* expression was suppressed but the *elovl5* expression was upregulated in the AA-*fads2−/−*testes (Figure 8C). Western blotting analysis indicated elevated synthesis of GJ marker Cx43 in the protein lysates of the testes of the nd- and AA-*fads2−/−* mice (Figure 8D), but suppression of TJ marker Claudin 11 (Figure 8E). Occludin synthesis was reduced in the DHA-*fads2−/−* testes (Figure 8F).

## 4 Discussion

This study elaborates the role of LC-and VLC-PUFAs as structural elements of the hydrophobic core of the membrane lipidomes of mouse ovaries and testes, indispensable for fertility. Auxotrophic, infertile, and PUFA-deprived *fads2−/−* mice were used to systemically remodel the membrane lipidomes via the sustained application of defined PUFA-supplemented diets. This study provides unbiased molecular proof of the principle of the pivotal role of PUFAs as nutritional essentials in remodeling the structure of the membrane lipidome of *fads2−/−* ovaries and testes. A sustained selected supply of nutrient EFA- (nd-), AA-, DHA-, and combined AA/DHA specifically modified membrane lipidomes of *fads2−/−* and PUFA-deficient ovaries and testes of which only the AA/DHA supplement fully reconstituted the fertility of the *fads2−/−* mice.

Replenishment *ab ovo* of the membrane lipidome of the *fads2−/−* ovaries via AA/DHA triggered the compaction of dissociated follicular GC layers in the *fads2−/−* ovaries and reconstituted the highly ordered GJ-Cx43 channel system and Cx37 assembly at the interphase between the GCs and ovum [14].

Reconstitution of the lipidome nd*-fads2*−/− testes via sustained AA/DHA diets *ab ovo* in pregnant *fads2+-* mothers and continued in *fads2−/−* male offspring after weaning remodeled the scaffold for the assembly of TJ, GJ, and desmosome protein complexes in the basolateral compartment of the SCs, thus restoring the BTB, which is essential for spermatogenesis and fertility.

Surprisingly, initiation of the sustained AA/DHA feeding regimen in 4-month-old adult *fads2−/−* female mice within four to six weeks triggered full restitution of oogenesis and the first complete ovarian cycle and regular spermatogenesis in the adult (4 months old) *fads2−/−*males, resulting in fertility and fecundity similar to control mating. This feeding period is required for the replenishment of PUFA patterns in the phospholipidomes [15].

Only the dietary supply of AA/DHA reconstituted the lipidomes of the ovaries and testes of the *fads2−/−* mice for regular ovarian cycles and spermatogenesis. The EFAs (nd) and ω6-AA- modified lipidomes of the *fads2−/−* mutant had no impact on *fads2−/−* infertility. Surrogate ω6-eicosatrienoic acid (20:3^5,11,14^) synthesized from linoleic acid in the nd-*fads2−/−* mice was unable to replace absent PUFAs.

These results add important data to our previous experiments [9] using cod liver oil as a DHA supplement of the nd diet of newborn *fads2−/−* mice. Cod liver oil supplies ω3-20:5 ^5,8,11,14,17^ (EPA) (17%) and ω3-22:6 ^4,7,10,13,16,19^ (DHA) (21%) in an approximately 1:1 M ratio. Our observation was at variance with a previous report that described a *fads2*−/− mouse mutant also characterized by impaired male reproduction and dermal and intestinal ulceration [10]. Male fertility of this mutant was recovered solely by DHA [16].

### 4.1 PUFAs in the regulation of gene and protein expression of oogenesis and spermatogenesis in the *fads2−/−* mice

Steady-state RNA concentrations of marker proteins of the ovaries and testes of the control and nd-*fads2−/−* mice during different developmental stages of folliculo- and spermatogenesis indicated significantly upregulated expression of *cx43*, *sf1*, and *jamC* in the nd*-fads2−/−* ovaries. The RNA levels of the two sperm tail-specific gene transcripts *tisp69* and *tisp76* [17] were suppressed in the nd- and AA*-fads2−/−* testes. *Sprm1*, a marker of terminal differentiating haploid spermatids, was highly expressed in the nd*-fads2−/−* testes. The reconstitution of spermatogenesis was convincingly documented by the topology of ACRV1, the marker of functional spermatozoa. ACRV1 was irregularly distributed in immature spermatocytes of the nd- and AA-*fads2*−/− but correctly assembled in the sperm heads of the DHA- and AA/DHA*-fads2−/−* males.

FADS1, FADS2, and elongases *elovl2* and *elovl5* are key players in AA and DHA synthesis from C18-EFAs and *elovl4* in the chain elongation of LC- to VLC-PUFAs [18,19]. The gene expression of *elovl4* was suppressed in the nd*-fads2−/−* testis. *Elovl5* was upregulated in the ovaries and testes of the AA*-fads2*−/− mice and *elovl 2* and *6* were suppressed in the AA*-fads2−/−* testes.

Plasma gonadal steroids testosterone and progesterone remained at or nearly the same level as in the control mice, which implied unimpaired regulation of the negative feedback on LH and FSH pituitary secretion*.*

### 4.2 A putative molecular view of ω3-and ω6-PUFAs in the GC and SC plasma membrane architecture

Numerous biophysical studies on lipid–lipid and lipid-protein model systems have contributed to the understanding of mammalian membrane structures [[20], [21], [22], [23]]. Studies on model systems of photoreceptor membranes [24,25] and pathogenetic studies on the mutated *elovl4* locus leading to VLC-PUFA deficiency in macular degeneration in Stargardt disease (STDT3) have considerably advanced the scope of PUFA function [26,27]. The understanding of the molecular function of LC-and VLC-PUFAs in the ovaries and testes is rather limited.

Current technologies preclude a sophisticated experiment-based molecular interpretation of the interactions of the lipid bilayer microenvironment and junction protein complex assemblies for GC connectivity and SC polarity and BTB. The detailed structural information on the lipidomes of the ovaries and testes of the control and *fads2−/−* mice, their targeted modification, and the impact on morphology and functional dynamics during folliculo-/oogenesis and spermatogenesis elaborated in this study warrants a speculative view of LC- and VLC-PUFA function in the architecture of the GC and SC membranes as illustrated in Figure SI3.

The lipidome of the testes of the nd*-fads2−/−* males was devoid of VLC-PUFA-substituted ceramides and sphingomyelin species. AA/DHA diet led only to the synthesis of ω6-28:4^13,16,19,22^ from AA, not to the VLC-PUFA pattern control testes mainly of ceramides and sphingomyelins substituted by normal and α-hydroxylated VLC-PUFAs 28:4 to 32:5 [[28], [29], [30], [31]].

The different lipid bilayer remodeling experiments demonstrated the requirement of AA and DHA for the reconstitution of the scaffold of the junction protein complexes and their dynamics in folliculo- and spermatogenesis. AA and EPA share identical chain lengths and a C-terminal polyene structure with the Δ5-cis double-bond position. ω6-20:4 is the only precursor in the biosynthesis of VLCPUFAs that explains the high efficiency of AA to its precursor function in the biosynthesis of abundant ω-28:4^13,16,19,22^ as ceramide substituent in the control and *fads2*−/− testes under AA/DHA dietary rescue.

Inactivation of the *elovl2* locus abrogates ω6-VLC-PUFAs synthesis causing male infertility, which is not overridden by ω3 DHA diets [32], but leaves female fertility unimpaired. The results from our *fads2−/−* and *elovl2−/−* mutants indicated the essential precursor role of AA in C28–C30-PUFA synthesis in spermatogenesis.

Furthermore, the absence of fucosyl-glycosphingolipids substituted with VLCPUFA discovered in germ cells of *galt−/−* testis leads to infertility [33].

The plasma membrane shows molecular asymmetry with the preferred topology of ceramides and derived sphingolipids, sphingomyelin, neutral glycosphingolipids, gangliosides, and cholesterol in the outer membrane lipid bilayer and PL classes PE, PI, and PS in the cytosolic inner leaflet of the bilayer [34,35]. Liquid-ordered detergent-resistant membrane domains (DIMS) or rafts in the outer lipid leaflet of the plasma membrane have been recognized as scaffold of multi- and single span proteins of TJs (claudins, occludin, and JAMs) and GJs (connexins) [[36], [37], [38], [39], [40], [41]]. Domain stability was maintained by specific molecular properties of its complex lipid constituents in the control gonadal membranes as schematically summarized in Figure SI3.

1. Polar head groups of phospholipids, sphingolipids, and cholesterol form a belt of hydrogen-bonding in the outer leaflet.
2. Saturated fatty acyl residues and saturated, ordered C12 to C18-carboxy domain of bipartite normal and α-hydroxylated VLC- PUFA-substituted sphingolipids spanning the outer leaflet.
3. Their polyunsaturated, highly disordered CH_3_ terminal polyene domains couples outer and inner membrane leaflets by interdigitating with PUFA-enriched DAG backbones of PE, PI, and PS in the cytoplasmic leaflets, well-known docking sites in signal transduction.
4. SM/C complexes stabilize cholesterol-rich DIM domains. Reduced cholesterol synthesis (Stoffel et al., 2014) shown in the *fads2*−/− mice together with the absence of LC- and VLC-PUFAs contribute to the disintegration and dissipation of the domain structural elements.

EM of the *fads2−/−* ovaries revealed markedly malformed and irregularly oriented TZPs. TZPs are specialized plasma membrane extensions of granulosa cells that project through the zona pellucida to the oolemma, where subunits of Cx43 of GC interact with subunits of CX37 in the oolemma. Dysfunction of these TZP-GJs is known to compromise oocyte growth and meiosis.

Future studies will reveal further details on the molecular interactions between nutritionally modified gonadal lipidomes and TJ and GJ function and the molecular interactions between specific ω3-and ω6-PUFA with integral membrane protein components of junction complexes in the plasma membrane of GCs and Sertoli cells in the regulation of oogenesis and spermatogenesis. The results of our study on the dietary control of fertility by ω3/ω6-PUFAs in the unbiased genetic mouse model warrant clinical trials on the extensively but controversially discussed role of PUFA in human nutrition improving fertility in both men and women, thereby contributing to improving national nutrition guidelines [42].

Finally, numerous studies demonstrated the impact of genetic backgrounds on LC-PUFAs biosynthesis and metabolism [[43], [44], [45], [46]].

The auxotrophy of the *fads2−/−* mutants suggests experimental time lapse mimicries of changing ω3/ω6-PUFA ratios from terrestrial (savannah) to maritime (seafood) web during evolution combined with the exploration of the proposed role as a nutritional epigenetic factor in evolution and population expansion [47].

## 5 Conclusions

Overall, we demonstrated in the unbiased PUFA synthesis-deficient auxotrophic *fads2*−/− mouse mutants in controlled feeding experiments biochemical, morphological, and functional evidence that highlight the pivotal structural role of two nutrient PUFAs, AA and DHA, as building blocks in the phosho- and sphingolipidomes of ovarian and testicular membranes for both female and male fertility.

## Author contributions

W·St. conceptualize the study, obtained its funding, participated in the investigation and supervision, wrote the original draft, and reviewed and edited the manuscript. I.S.S., B.J., and E.B. participated in the investigation and validated the data. A. Thomas and M. Thevis participated in the investigation. I.W. participated in the investigation and validated the data, produced the art work, and helped write and edit the manuscript.
