# Supplementary material for: Dietary ω3-and ω6-Polyunsaturated fatty acids reconstitute fertility of Juvenile and adult Fads2-Deficient mice
Source: Mol Metab. 2020 Mar 17;36:100974. doi: 10.1016/j.molmet.2020.100974 (PMC7153284; doi:10.1016/j.molmet.2020.100974)
Supplement: Multimedia component 1 [file mmc1.docx]

**Supplemental Information**

**Dietary ω3- and ω6-polyunsaturated fatty acids reconstitute fertility of juvenile and adult FADS2-deficient female and male mice**

Wilhelm Stoffel*^,1, 2, 3^, Inga Schmidt-Soltau^2^, Erika Binczek^1^, Andreas Thomas^4^, Mario Thevis^4^, Ina Wegner^1^

^1^Laboratory of Molecular Neuroscience, Institute of Biochemistry, University of Cologne, 50931 Cologne, Germany

^2^CMMC (Center for Molecular Medicine), Faculty of Medicine, University of Cologne, 50931 Cologne, Germany

^3^CECAD (Cluster of Excellence: Cellular Stress Responses in Aging-Associated Diseases), University of Cologne, 50931 Cologne, Germany

^4^Institute of Biochemistry, Deutsche Sporthochschule Cologne, 50933 Cologne, Germany

* Corresponding author

Wilhelm Stoffel, MD, PhD , Laboratory of Molecular Neuroscience, Institute of Biochemistry, University of Cologne, 50931 Cologne, Germany

* Correspondence

e-mail: wilhelm.stoffel@uni-koeln.de

phone +49-221-478-6881

fax +49-221-478-6882

**Diets**

Altromin diet #1310 was the basic diet (nd), which contains the two essential fatty acids (EFA) 18:2 and α-18:3 in concentrations to prohibit EFA deficiency. Arachidonicacid (AA) was supplemented as ARASCOand docosahexaenoicacid (DHA) as DHASCO trigycerides. These PUFAs are the only PUFA in the respective supplemented nd-diet,. Dietary fat content contributes 14% (468 kcal/kg) of total metabolic energy (3342 kcal/kg). AA contributes 1.2% and DHA 1.1% of total metabolic energy, respectively.

**Table SI 1**

Fatty acid composition of lipid extracts of respective diets

| diet | nd | ω6-AA | ω3-DHA | ω6AA/ω3DHA |
| --- | --- | --- | --- | --- |
| FA | % | % | % | % |
| 16:0 | 26 | 13 | 12 | 11 |
| 18:0 | 3 | 2 | 2 | 2 |
| 18:1 | 34 | 24 | 24 | 22 |
| 18:2 | 37 | 55 | 56 | 53 |
| ω6-20:4 |  | 6 |  | 6 |
| ω3-22:6 |  |  | 6 | 6 |

**Primer list**

| tisp69 s | 5'-cctggccgcatctacgctgttcgtgctgcg-3' |
| --- | --- |
| tisp69 as | 5'-tcaggagagctcggaagtcgcttctgactg-3' |
| tisp76 s | 5'-cagggctttgtgcctgaagacagaccactg-3' |
| tisp76 as | 5'-ccacctctttcagcagctcttcctccacag-3' |
| ar s | 5'-gacagtgccaaggagttg-3' |
| ar as | 5'-gagctacctgcttcactg-3' |
| sox9 s | 5'-ctacccgcccatcacccgctcgcaatacga-3' |
| sox9 as | 5'-ctgtgtgtagactggttgttcccagtgctg-3' |
| vimentin s | 5'-caggtctgtgtcctcgtc-3' |
| vimentin as | 5'-cagctcctgcagttctac-3' |
| cnx43 s | 5'-accaacggccccactctcacctatgtctcc-3' |
| cnx43 as | 5'-taaatctccaggtcatcaggccgaggtctg-3' |
| sprm1 s | 5'-ctatgtttgggaaggttctcagccagacga-3' |
| sprm1 as | 5'-agttctctgacttagtgagggtgggagtgg-3' |
| prm1 s | 5'-gacccctgctcacaggttggctggctcga-3' |
| prm1 as | 5'-attggcaggtggcattgttccttagcaggc-3' |
| occludin s | 5'-ctgggtcagggaatatccacctatcacttc-3' |
| occludin as | 5'-gcaactggcatctctctaaggtttccgtct-3' |
| claudin1 s | 5'-gtgcagaagatgtggatggctgtcattggg-3' |
| claudin1 as | 5'-gatctcttcctttgcctctgtcacacatag-3' |
| claudin11 s | 5'-gctgcctccgttatgggcctgcccgccatc-3' |
| claudin11 as | 5'-ttagacatcggcactcttggcatgcgttgg-3' |
| jamA s | 5'-agccagatcacagctcccta-3' |
| jamA as | 5'-tggatggaggtacaagcaca-3' |
| jamB s | 5'-gagtggaagaaggtgggaca-3' |
| jamB as | 5'-aggaacagcaggagccacta-3' |
| jamC s | 5'-gaactcggagacaggcactc-3' |
| jamC as | 5'-cgtctgtacgcacagcagat-3' |
| sf1 s | 5'-ggctgcagggctgcaggggtttataagggc-3' |
| sf1 as | 5'-gaaagtgtgtgagagagagtgggcaggag-3' |
| hgprt s | 5'-gctgacctgctggattacattaaagcactg-3' |
| hgprt as | 5'-attcctgaagtactcattatagtcaagggc-3' |


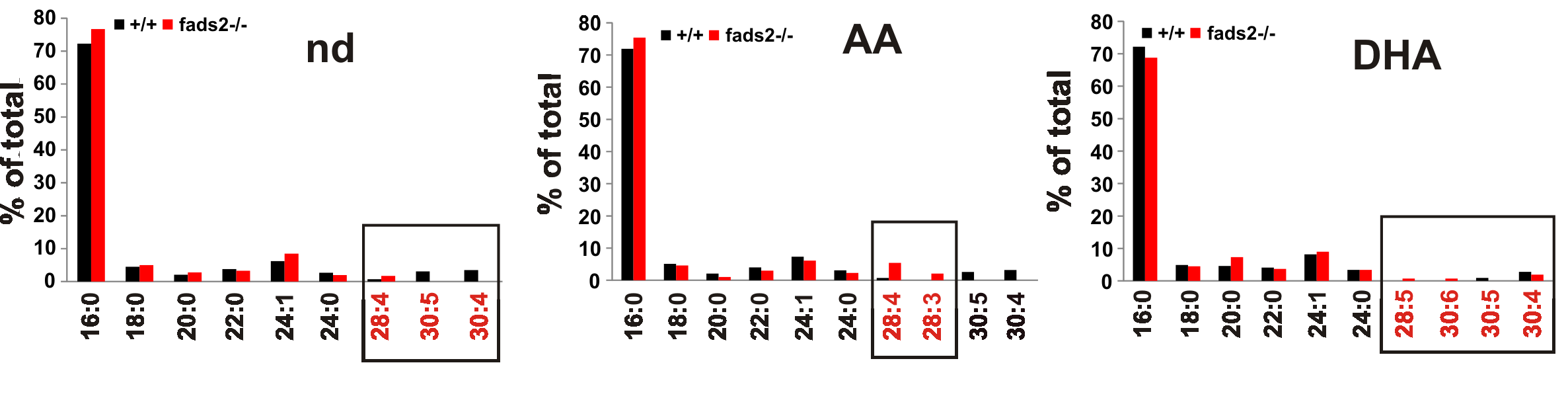


**Figure SI 1** GC/MS- analysis of fatty acids pattern of sphingomyelin in testicular lipid extract of nd-, AA- and DHA-fads2-/- mice

**Sperm analysis**

Epididymes of adult male nd-, AA-, DHA, and AA/DHA-mice were isolated and dissected free of epididymal fat. The cauda was opened by a radial slit, transferred to microcentrifuge tubes containing 1x PBS and shaken for 10 min. Epididymal tissue was allowed to settle for 10min. Sperm suspension was removed for count, motility and purity check and pelleted at 15000xg for 10 minutes. Aliquots of the suspension were used for sperm count and spreading on slides for microscopy (“smears”). Slides were incubated in 10mM DTT, 0,1M Tris pH8 buffer for 5-10 minutes, followed by a 2 hour incubation in 10mM DTT, 10mM lithium diiodo-salicylate, 0,1M Tris pH 8, fixed in 4% PFA for 1 hour, washed with PBS and permeabilized in 2% Triton X-100, 0,1% BSA in PBS for 15 minutes. Smears were washed with PBS, blocked with 3% BSA, 0,1% Tx100 in PBS and incubated with anti-Acrosomal vesicle protein 1 (ACRV1) antibody (antibodies online, Aachen, Germany) in blocking buffer for 1hour, washed three times for 10 minutes with PBS and developed with secondary antibody for 2 hours

**Protein analysis**

**Ovary and testis** of wt and *fads2-/- fe*male and male mice were homogenized in lysate buffer containing the protease inhibitor cocktail Complete (Roche, Penzberg, Germany) with an Ultra Turrax. Protein concentrations were measured using the PIERCE BCA protein assay kit (Thermo Fisher Scientific, Darmstadt, Germany).

**Protein aliquots (100µg) were separated by NuPAGE, 4-12% BIS-TRIS gels and transferred to a Nitrocellulose membrane, using the NuPAGE Western Blot system (Invitrogen,** Darmstadt, Germany**). Blots were immuno-stained overnight at 4°C with respective antibodies:** anti-Occludin (Thermo Fisher Scientific, Darmstadt, Germany), anti-Claudin11(Santa Cruz, Heidelberg, Germany) and anti-Cx43 (Thermo Fisher Scientific, Darmstadt, Germany). After washing horseradish peroxidase conjugated secondary antibodies were used and detected with the ECL system. **Signals were quantified by densitometry using the ImageJ2 program (**RRID:SCR_003070)**.**

**Histology and Immunohistochemistry**

Mice were perfused from the left ventricle with 25ml PBS and with 50ml PBS-buffered 4% paraformaldehyde for paraffin embedding and processing for immunofluorescence-microscopy. Sections were permeabilized with 0.5% Triton X-100/PBS at 4°C, blocked with 3% BSA/0.1% Triton X-100/PBS and treated with respective antibody dilutions in 3% BSA/0.1% Triton X-100/PBS, at 4°C over-night: anti-Occludin (Thermo Fisher Scientific, Darmstadt, Germany), -Claudin11 (Santa Cruz, Heidelberg, Germany), -ZO1 (Invitrogen, Darmstadt, Germany), -JAM-A and -JAM-C (kindly provided by Dr. Ebnet, Münster, Germany), -Wnt3a (Santa Cruz, Heidelberg, Germany), -ß-Catenin (BD Transduction, Heidelberg, Germany), -Cx37(Thermo Fisher Scientific, Darmstadt, Germany) and -Cx43 (Thermo Fisher Scientific, Darmstadt, Germany) antibodies. Sections were washed twice with PBS/0,05% Tween20, incubated with Cy3-conjugated 2. IgG antibody (Jackson Immuno Research) for 1h at 37°C, and immuno-stained with affinity purified rabbit polyclonal or monoclonal antibodies horseradish-peroxidase with DAB Substrate, (Roche #1718096) in the peroxidase reaction. A Zeiss microscope Axio Imager, M1 and the AxioVision Imaging Software (RRID:SCR_002677) were used for fluorescence microscopy and a Slide Scanner Leica SCN400 and the Aperio ImageScope Software (RRID:SCR_014311) for peroxidase microscopy.

**Serum testosterone and progesterone quantitation by liquid chromatography-mass spectrometry (LC-MS)**

The steroid analysis was performed using liquid chromatography (Waters Aquity UHPLC, Eschborn, Germany) coupled to mass spectrometry (Waters TQXS, Eschborn, Germany). A C8 analytical column (Poroshell C8, 3x50 mm, 2,7 µm particle size, Agilent, Waldbronn, Germany) was used for liquid chromatography A gradient using buffer A (5 mM ammonium acetate, pH 3.5) and buffer B (methanol) was started with an isocratic step for 0.5 min at 40 % of buffer B, increased to 95 % of buffer B in 10 minutes, and re-equilibrated at starting conditions for 2 minutes. The overall run time was 12 minutes with a flow rate of 0.3 ml/min.

The mass spectrometer operated in positive MRM mode and unispray ionization. Impactor voltage was adjusted to 0.6 kV. Diagnostic ion transitions at *m/z* 289-97, 289-109 (T), 315-97, 315-109 (P), 292-97, 292-109 (IS1), 324-100, 324-112 (IS2) were monitored for Testosterone (T), Progesterone (P), ^2^H_3_-Testosterone (IS1) and ^2^H_9_-Progesterone (IS2). Argon was used as collision gas and collision energies were optimized at 21, 28 (T), 23, 26 (P), 23, 23 (IS1), 20 and 28 (IS2) eV. The dwell time for each transition was fixed at 0.012 s and the cone voltage ranged between 30 and 48 V.


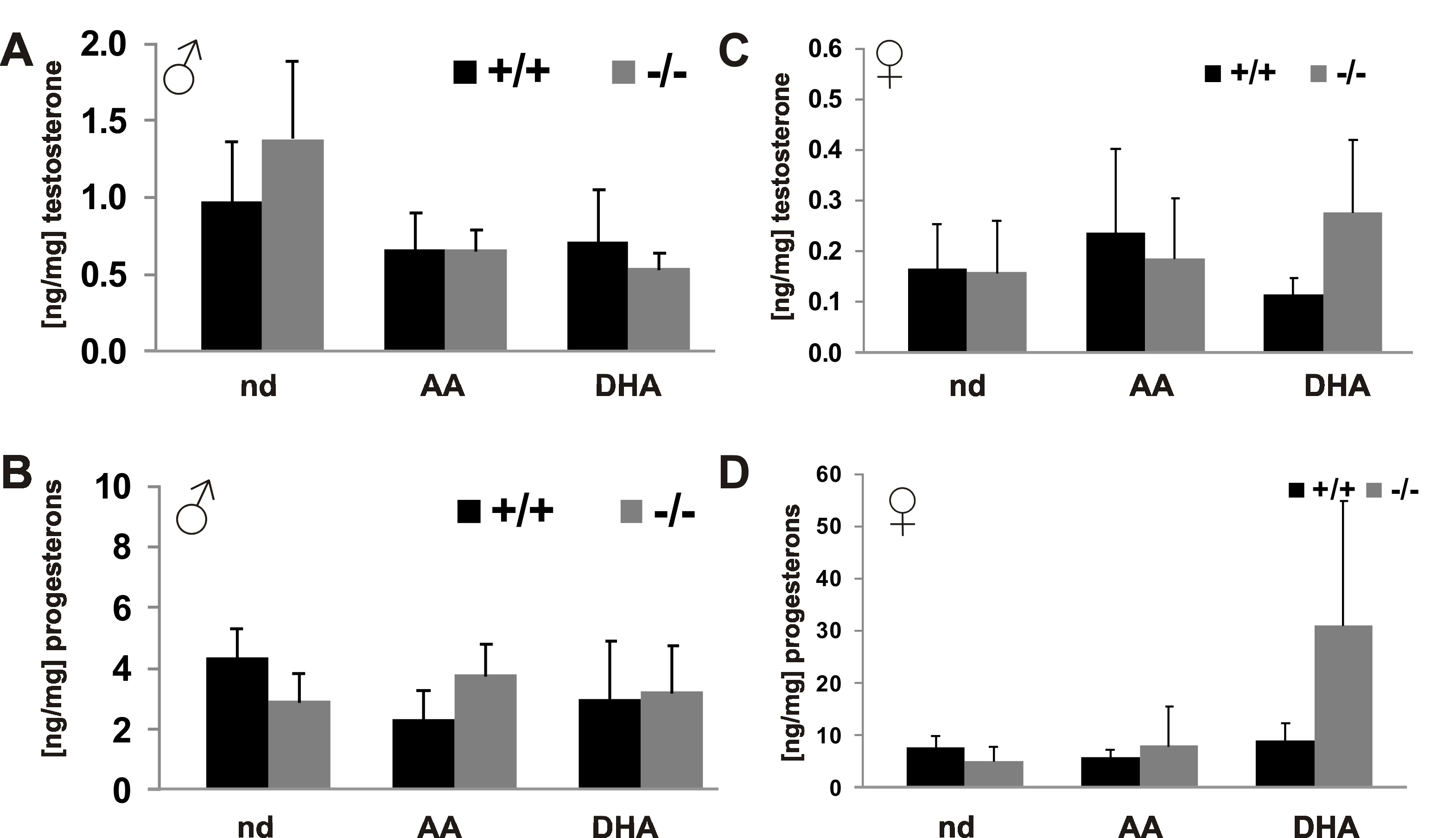


**Figure SI 2 Serum testosterone and progesterone concentrations in cohorts (n=8) of nd-, AA- and DHA-*fads2-/-* male and female mice.**

**Lipid analysis**

An essential prerequisite of lipid analysis was that all analytical steps, lipid extraction, HPTLC, MS/MS, saponification and transesterification of fatty acid methylesters by combined gas-liquid-chromatography -mass spectrometry (GC-MS) were carried out under argon or nitrogen.

**Phospho- and sphingo-lipidome analysis**

Total lipids were extracted from ovary, testis and epidydimis by homogenizing tissue in an Ultraturrax in 10 volumes of chloroform/methanol C/M 2:1 (v/v), and re-extracted using C/M 1:1 (v/v) and then C/M 1:2 (v/v) for 1 h each at 37°C under a stream of nitrogen. The combined extracts of total lipids were dissolved in C/M 2:1 (v/v), washed with 2 M KCl, and concentrated for separation by HPTLC, using HPTLC plates (Merck, Germany) in solvent system chloroform/ethanol/triethylamine/water 60/70/70/14 (v/v/v/v) in. Bands were identified by primuline (2mg/dl acetone-water 5:1) and by Zinzadze reagent for phospholipids, anthrone reagent for carbohydrate-containing sphingolipids, and by charring with 50% H2SO4/H2O for all lipid classes. Phospho- and sphingolipid classes were separated by HPTLC, and after elution, species of PL-classes analyzed by MS/MS using an Applied BiosystemsQTrap 2000 mass spectrometer (Darmstadt, Germany). Analytes were dissolved in methanol/5mM ammonium acetate and injected into the ion source by means of a Hamilton syringe pump at 3μL/min. The ESI source was operated at room temperature using a spray voltage of 5500V. Nitrogen was used as the sheath and collision gas and was obtained from a Peak Scientific (Inchinnan, Scotland) MN30L nitrogen generator. Declustering and collision offset potentials were adjusted to 50V in precursor as well as product ion scan experiments. A mass range of 200-750 u and 50-700 u was recorded, respectively. The mass spectrometer was operated in the positive mode using an electrospray voltage of 5500 V and multiple reaction monitoring (MRM).

**Fatty acid profiling of phospho- (PL) and sphingolipid-classes(SL) by GC-MS**

PL and Sl-bands visualized with primuline, were collected on small-fritted glass filters and eluted with C/M 2:1(v/v)into Sovirel glass-tubes for extraction and further fatty acid analysis.

Ester lipids were saponified in0.5NKOH in methanol for 2 h at 40°C. Unsaponifiable lipids were extracted with dichloromethane, the aqueous phase was acidified with 2 N HCl, and fatty acids were extracted with hexane/ether 1:1 (v/v), concentrated, and esterified with 5% HCl-methanol at 80°C for 1 h. One volume of water was added and fatty acid methylesters (FAME) extracted with hexane and concentrated under nitrogen for GC-MS and GC analysis.

PL- and Sl-classes were transesterified and the fatty acid profiles determined by combined gas-liquid-chromatography -electrospray ionization mass spectrometry (ESI-MS) on an Agilent (Waldbronn, Germany) 6890/5973N instrument equipped with a HP-5MS fused silica column (length 17 m, i.d. 0.25 mm, film thickness 0.25 μm) or on a Carlo Erba Instrument Model GC8000.

Samples of fatty acid methylesters were injected in a 10:1 split mode into the mass spectrometer, which was operated in full scan mode over a mass range of 50-500 u, and electron ionization (EI) was utilized at 70 eV. The injector temperature was set to 300°C. The gradient: 140°C – ramp1 10°C/min - 240°C – ramp2 20°C/min -300° - hold time 3minC , used allowed base line separation of all ω3- and ω6-PUFA-methylesters.


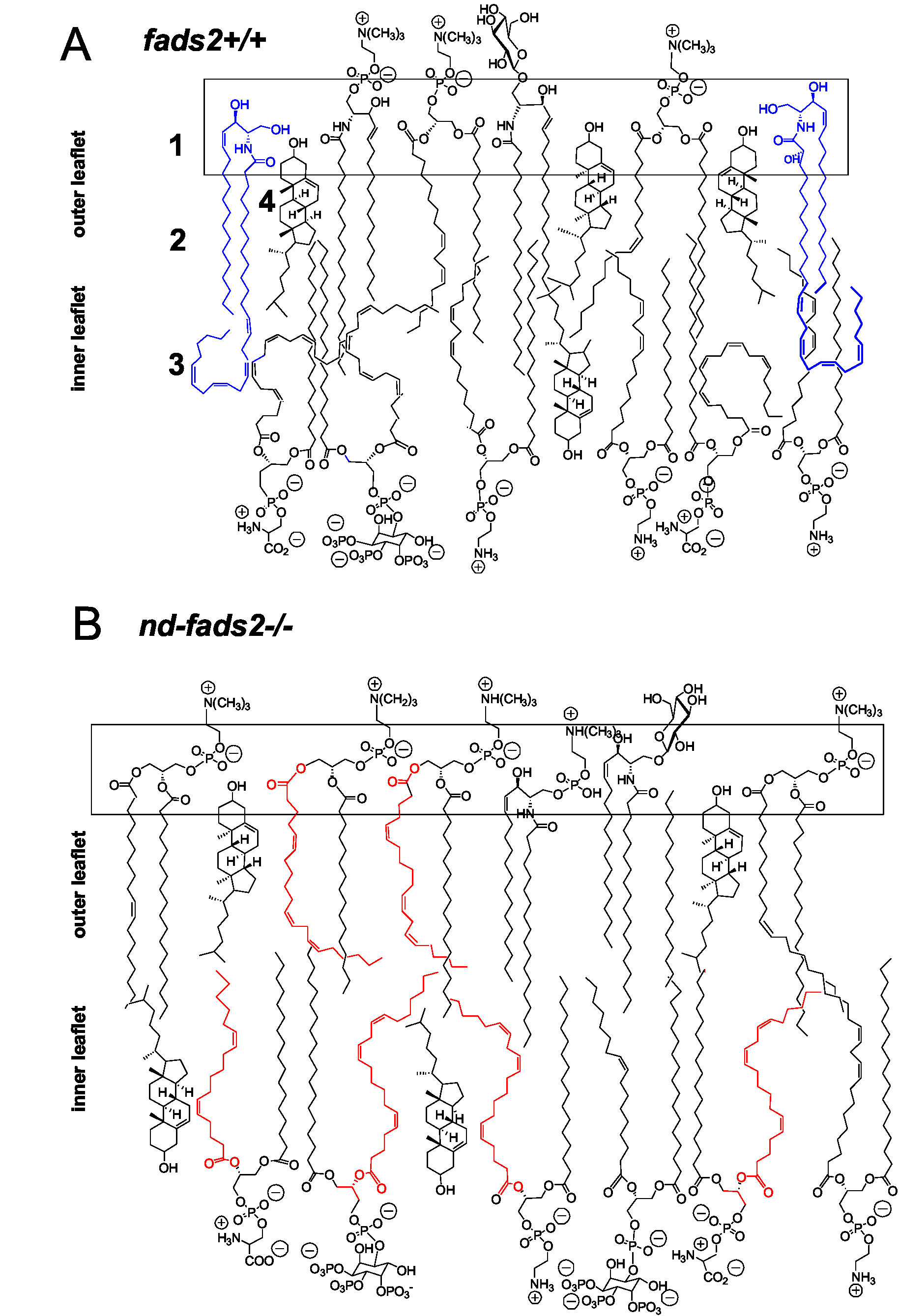


**Figure SI3** Schematic overview of structures illustrating asymmetric assembly of the lipidomes of outer and inner membrane leaflets of DIMs of Sertoli cells of (A) *control* and (B) *fads2-/-* testis. (A, B) Hydrogen-bonding belts at the interface are encased. (B) Surrogate 20:3^5,11,14^ acyl chains in the phospholipidome of *nd- fads2-/-* testis are highlighted (red), (A) VLC-PUFA-substituents of ceramides of control testis (blue). (1) hydrogen---bonding belt, (2) saturated hydrophobic chains, (3) CH_3_-terminal polyene domain of VLC-PUFAs, (4) cholesterol-sphingolipid-complex
